# Classification of Mobile-Based Oral Cancer Images Using the Vision Transformer and the Swin Transformer

**DOI:** 10.3390/cancers16050987

**Published:** 2024-02-29

**Authors:** Bofan Song, Dharma Raj KC, Rubin Yuchan Yang, Shaobai Li, Chicheng Zhang, Rongguang Liang

**Affiliations:** 1 Wyant College of Optical Sciences, The University of Arizona, Tucson, AZ 85721, USA; 2 Computer Science Department, The University of Arizona, Tucson, AZ 85721, USA

**Keywords:** Vision Transformer, Swin Transformer, oral cancer, oral image analysis, artificial intelligence

## Abstract

**Simple Summary:**

Transformer models, originally successful in natural language processing, have found application in computer vision, demonstrating promising results in tasks related to cancer image analysis. Despite being one of the prevalent and swiftly spreading cancers globally, there is a pressing need for accurate automated analysis methods for oral cancer. This need is particularly critical for high-risk populations residing in low- and middle-income countries. In this study, we evaluated the performance of the Vision Transformer (ViT) and the Swin Transformer in the classification of mobile-based oral cancer images we collected from high-risk populations. The results showed that the Swin Transformer model achieved higher accuracy than the ViT model, and both transformer models work better than the conventional convolution model VGG19.

**Abstract:**

Oral cancer, a pervasive and rapidly growing malignant disease, poses a significant global health concern. Early and accurate diagnosis is pivotal for improving patient outcomes. Automatic diagnosis methods based on artificial intelligence have shown promising results in the oral cancer field, but the accuracy still needs to be improved for realistic diagnostic scenarios. Vision Transformers (ViT) have outperformed learning CNN models recently in many computer vision benchmark tasks. This study explores the effectiveness of the Vision Transformer and the Swin Transformer, two cutting-edge variants of the transformer architecture, for the mobile-based oral cancer image classification application. The pre-trained Swin transformer model achieved 88.7% accuracy in the binary classification task, outperforming the ViT model by 2.3%, while the conventional convolutional network model VGG19 and ResNet50 achieved 85.2% and 84.5% accuracy. Our experiments demonstrate that these transformer-based architectures outperform traditional convolutional neural networks in terms of oral cancer image classification, and underscore the potential of the ViT and the Swin Transformer in advancing the state of the art in oral cancer image analysis.

## 1. Introduction

Oral cancer is a serious global health challenge, and its incidence continues to rise, posing a major threat to public health. According to the World Health Organization (WHO) [1], oral cancer is one of the ten most common cancers worldwide, with an estimated 377,713 new cases to be reported in 2020 alone [2]. Despite advances in medical research and technology, the prognosis for oral cancer patients remains challenging, largely due to late diagnosis [3]. Early detection remains a key factor in improving survival rates and reducing the burden of oral cancer on patients and healthcare systems [4]. Traditional methods of detecting oral cancer include clinical examination and, in some cases, invasive biopsy. However, these methods are not without limitations. In the search for non-invasive, cost-effective, and convenient screening methods, mobile imaging devices are being explored as a potential game changer in the field of oral cancer diagnosis [5]. Often embedded in smartphones, these devices provide a cost-effective and easy-to-use means of capturing high-quality images of the oral cavity. The use of mobile devices for imaging provides a unique opportunity for early detection and remote screening. The widespread availability of smartphones makes this approach particularly advantageous for large-scale screening programs, especially in resource-limited settings where traditional diagnostic infrastructure may be lacking. However, the large amount of image data generated by mobile devices poses a serious challenge [6]. Efficiently and accurately analyzing these images requires advanced computational methods, which set the stage for integrating artificial intelligence into the field of oral cancer detection.

Artificial intelligence has showcased remarkable effectiveness across diverse domains, with a notable impact in medical image analysis [7,8]. Convolutional neural networks (CNNs) have traditionally been at the forefront of image classification tasks, excelling in identifying abnormalities and diseases in medical images [9,10,11]. However, a paradigm shift has occurred with the emergence of transformer-based architectures [12]. The transformer architecture is built upon a self-attention mechanism, enabling the model to learn intricate relationships between sequence elements. While attention models have been widely employed in feed-forward and recurrent networks, transformers uniquely rely entirely on the attention mechanism [13]. Vision transformers (ViTs) [14] have multiple advantages compared to CNNs for vision tasks. ViTs [15] capture global dependencies in the input image by considering all image segments simultaneously. This helps to understand the relationships between different parts of the image and is beneficial for tasks that require global context. ViTs use a self-attention mechanism that allows them to weigh the importance of different image segments during the learning process. This attentional mechanism provides interpretability, allowing researchers to understand which parts of an image are more critical for making predictions.

ViTs have been shown to drive state-of-the-art technology in a wide range of vision tasks, including image classification [16], object detection [17], semantic segmentation [18], image colorization [19], and video understanding [20]. ViTs have garnered great interest in the medical imaging community and have been used in multiple medical imaging applications. Costa et al. [21] used ViT with performer to classify lung CT images for COVID-19 diagnosis and achieved good performance. Tanzi et al. [22] applied a Vision Transformer (ViT) for femur fractures classification with X-ray images, and outperforming the state-of-the-art approaches based on CNN, the attention maps and clustering further showed the reliability of the approach. Gheflati et al. [23] applied Vision Transformers (ViT) to categorize breast ultrasound images, revealing that ViT models demonstrate efficiency comparable to, or even surpassing, CNNs in the classification of ultrasound breast images. This underscores the significant potential of ViT models in the realm of breast ultrasound image classification. In a distinct study, Jiang et al. [24] devised an ensemble model integrating the Vision Transformer model and the EfficientNet model into the ViT-CNN ensemble model for diagnosing acute lymphoblastic leukemia. The results exhibited a noteworthy superiority of the ViT-CNN ensemble model over three classic convolutional neural network models. Chen et al. [25] introduced TransUnet, a model incorporating both transformers and Unet for medical image segmentation. Their investigation demonstrated that transformers function as robust encoders for medical image segmentation tasks. The amalgamation with Unet enhances finer details by recovering localized spatial information, leading to superior performance compared to various competing methods across diverse medical applications. In a separate contribution, Chen et al. [26] presented ViT-V-Net, combined Vision Transformers and ConvNets, designed for volumetric medical image registration. Experimental results showed the superior performance of the proposed architecture when compared to several top-performing registration methods.

The Swin Transformer [27,28] is a transformer-based model with state-of-the-art performance in vision tasks. It is highly efficient and accurate, and outperformed multiple existing transformer-based models on number of benchmark datasets and tasks. The Swin Transformer is used as the backbone for many vision-based model architectures due to these desirable properties. The Swin Transformer uses a combination of local and global attention mechanisms to process images and improve accuracy, it uses a series of shifted window attention mechanisms that enable the model to focus on different parts of the image at different scales, and a hierarchical structure that enables the model to learn and reason about the relationships between different image regions. Zhang et al. [29] present a deep learning-based framework for the diagnosis of COVID-19 using chest CTs with a Unet and Swin Transformer backbone and achieved good results. Xie et al. [30] proposed the Swin-SimAM network. They incorporated the SimAM attention module, which is parameter free, to emphasize crucial parts of skin lesions for improved melanoma detection. Additionally, the Swin Transformer was applied to medical segmentation tasks. Hatamizadeh et al. [31] introduced the Swin Unet Transformer, utilizing a U-shaped network which used the Swin Transformer as the encoder, and achieved good performance for semantic segmentation of brain tumors in MRI images. In this study, we utilized the Vision Transformer and the Swin Transformer for mobile-based oral cancer image classification. The pre-trained Swin Transformer model exhibits notable performance, achieving an accuracy of 88.7% in the binary classification task, this surpassed the ViT model by 2.3%. And both transformer-based models outperformed the classic CNNs. The experimental results showed the promising potential of the Vision Transformer and the Swin Transformer in pushing the boundaries of oral cancer image analysis and advancing the state of the art in this field.

## 2. Materials and Methods

### 2.1. The Vision Transformer

**Figure 1:**
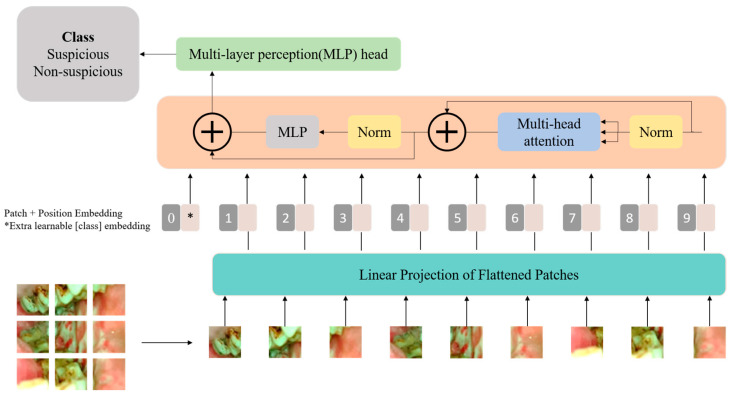
The block diagram of the Vision Transformer architecture used in this study.

Visual transformers (ViTs) are a pioneering approach in the field of computer vision that challenges traditional convolutional neural networks (CNNs) in image processing tasks. Visual transformers are an extension of the transformer architectures originally designed for natural language processing and have achieved significant success in various computer vision benchmark tests [15]. Unlike traditional CNNs, ViTs rely on a pure transformer architecture. The architecture of the Vision Transformer used in this study is shown in Figure 1.

A critical aspect of Vision Transformers is the self-attention mechanism, which allows the model to weigh the importance of different patches when processing a particular patch. This mechanism enables the model to capture long-range dependencies and contextual information, making it highly effective for image understanding. The self-attention mechanism computes attention scores between all pairs of positions in the input sequence, generating an attention matrix. This matrix is then used to weigh the importance of each patch during the aggregation of information. The ability to attend to different regions of the image simultaneously enhances the global context awareness of Vision Transformers. The input of the self-attention block is a sequence of embeddings representing different positions or tokens in the input sequence, and each embedding corresponds to a position in the input sequence. The embeddings are linearly transformed into three vectors for each position, key, query, and value, these transformations are learned during the training process of the Vision Transformer. The output of a self-attention block in a Transformer is a weighted sum of the input embeddings, determined by attention scores that reflect the relationships between different positions in the input sequence. Each input embedding undergoes linear transformations to obtain query, key, and value vectors, and attention scores are computed by taking the dot product of query and key vectors. These scores are then normalized using the softmax function, producing weights that represent the relevance of each position. The output captures the contextual information from the entire input sequence, with each position attending to other positions based on their relevance.

The multi-head self-attention mechanism in a Vision Transformer (ViT) is a key component that enhances the model’s ability to capture diverse patterns and relationships in visual information. The outputs from these parallel attention heads are then concatenated and linearly transformed to produce the final multi-head attention output. The use of multiple attention heads allows the model to focus on different aspects of the input sequence, enabling it to capture both fine-grained and coarse-grained features effectively. The multi-head mechanism enhances the model’s representational capacity and is a crucial element in the success of Vision Transformers across various computer vision tasks. Self-attention and multi-head self-attention can be mathematically expressed as follows. *W^Q^*, *W^K^*, *W^V^* are the learned weight matrices for the query (*Q*), key (*K*), and value (*V*) transformations.

$$Self\;Attention\left(Q,K,V\right)=softmax(\frac{QK^{T}}{\sqrt{d_{q}}})V$$

$$Multi\;Head\left(Q,K,V\right)=Concat\left({head}_{1},\ldots {head}_{h}\right)W^{O}$$

$${head}_{i}=Attention\;(QW_{i}^{Q},KW_{i}^{K},VW_{i}^{V})$$

The output from the multi-head self-attention block is inputted into a point-wise feed-forward network (*FFN*) that incorporates two linear activation functions and a rectified linear unit (*ReLU*) activation. *X* represents the output of the previous layer and the weight matrices of the first and second linear layers as *W_a_* and *W_b_*, and the bias vectors as *B_a_* and *B_b_*. The output of the point-wise feed-forward network can be expressed mathematically as:

$$FFN=ReLU\left(XW_{a}+B_{a}\right)W_{b}+B_{b}$$

*ReLU* is a common non-linear activation function that introduces element-wise non-linearity to the model. This process allows the model to capture complex and non-linear patterns specific to each position independently. The output of the point-wise FFN contributes to the enriched representation of each position in the ViT, enabling the model to learn and represent intricate features in the input image sequence.

For training and inference with Vision Transformers (ViTs), the input image is partitioned into a sequence of non-overlapping fixed-size patches. Each patch undergoes linear embedding, transformed into a flattened vector through a trainable linear transformation. This positional information, in conjunction with the sequence of overlapping patches, is then introduced into the encoder block of the transformer, allowing the model to process and capture both spatial and contextual information from the image in a sequence format.

### 2.2. The Swin Transformer

Vision Transformers process images by dividing the images into a sequence of fixed-size non-overlapping patches. However, this approach may not fully capture the intricate details of an image, especially when dealing with a combination of local and global features. The Swin Transformer has several advantages over ViTs and addresses some limitations of ViTs. The Swin Transformer introduced a hierarchical structure and shifted windows, providing a more efficient way to capture spatial hierarchies and local-global relationships within images. ViTs utilized a flat structure, which may hinder their ability to understand complex patterns in visual data. In addition, ViTs exhibit quadratic complexity, which poses computational challenges, especially for high-resolution images, while Swin Transformers alleviate this by introducing a more efficient hierarchical structure.

Similar to ViT, the process of the Swin Transformer begins by dividing the input image into distinct, non-overlapping patches through a dedicated patch splitting module. Treating each patch as a ‘token’, its feature is composed by concatenating the raw pixel RGB values. A linear embedding layer is then applied to project these raw-valued features into an arbitrary dimension denoted as C. A series of transformer blocks, featuring modified self-attention computation known as Swin Transformer blocks, is subsequently employed on these patch tokens. These transformer blocks, keeping the initial number of tokens intact, form ‘Stage 1′ alongside the linear embedding. To establish a hierarchical representation, a patch merging layer is utilized to reduce the number of tokens as the network advances deeper. The initial patch merge layer combines the features of each 2 × 2 neighboring patch group, applying a linear layer over the resulting 4C-dimensional concatenated features, resulting in a 4-fold reduction in the number of tokens. This initial patch merging and feature transformation block is denoted as ‘Stage 2’. This process repeats twice, creating ‘Stage 3′ and ‘Stage 4’.

The Swin Transformer introduces a novel shifted window-based self-attention mechanism. This approach aims to adeptly capture both local and global features, different from the conventional multi-head self-attention (MSA) model typically found in traditional transformer blocks. The standard Transformer architectures for vision tasks employ a global self-attention mechanism that involves computing relationships between a token and all other tokens. This global computation results in quadratic complexity relative to the number of tokens, making it unsuitable for many vision tasks that demand an extensive set of tokens for dense prediction or for presenting high-resolution images.

The primary aim of the shifted window is to execute self-attention within localized windows. Each window is composed of non-overlapping patches with dimensions MXM, and self-attention is computed within this window. As a result, there is a reduction in computational complexity; while the original multi-head self-attention (MSA) exhibits quadratic complexity concerning the patch number, the window-based MSA demonstrates linear complexity.

The Swin Transformer integrates a shifted window partitioning strategy, alternating between two configurations across consecutive blocks to efficiently model window connections. The initial module employs a standard window configuration, allowing for local self-attention computation from evenly spaced windows, commencing from the top-left pixel. Subsequently, the subsequent Swin Transformer block adopts a window configuration shifted by (M/2, M/2) pixels from the preceding layer. This strategic shift contributes to the model’s capacity to capture diverse spatial relationships effectively. Self-attention of Swin transformer blocks can be mathematically expressed as follows, where *B* is a relative position bias of the window.

$$Self\;Attention\left(Q,K,V\right)=softmax(\frac{QK^{T}}{\sqrt{d_{q}}}+B)V$$

**Figure 2:**
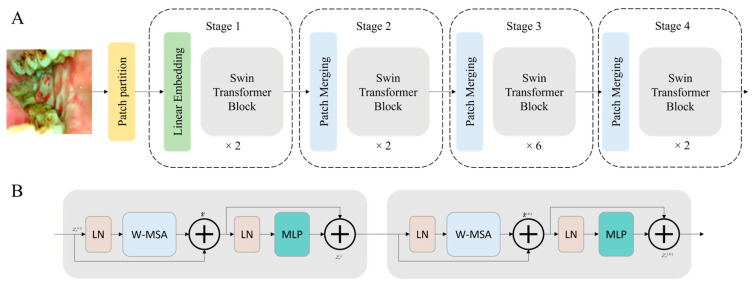
The block diagram of the Swin Transformer architecture (**A**) and the Swin Transformer blocks (**B**) that are used in this study.

ViT and Swin transformer architectures have several variants; in this study, we used the base models, ViT-B and Swin-B. The architecture of the Swin Transformer used in this study is shown in Figure 2.

### 2.3. CNN Models for Comparison

We used two classic CNN models VGG19 [32] and ResNet50 [33] for comparison. These two models were also pre-trained with the ImageNet dataset.

## 3. Experiments and Results

### 3.1. The Dataset

The dataset employed in this study was obtained through our customized oral cancer screening platform [34,35,36], utilized in the outpatient clinics of the Department of Oral Medicine and Radiology at the KLE Society Institute of Dental Sciences (KLE), the Head and Neck Oncology Department of Mazumdar Shaw Medical Center (MSMC), and the Christian Institute of Health Sciences and Research (CIHSR) in India. Every participant in this study received direct telediagnosis by specialists remotely [35]. The details of the oral cancer screening study regarding the image quality assessment and standardization, and software (including mobile application and cloud server) have been described in our previous published paper [35].

For this study, we used a total of 2434 oral images. The images were separated into two categories: ‘non-suspicious (1243 images), which contains normal and benign mucosal lesion images, and ‘suspicious’ (1191 images), which contains oral potentially malignant lesion (OPML) and malignant lesion images. The oral images were annotated by oral oncology specialists.

### 3.2. Data Augumentation

The efficacy of a network’s representational capacity is closely related to the amount of training data, especially for the transformer models. Generally, a larger dataset correlates with stronger representation ability of the model, and improved classification performance. Data augmentation refers to the method of introducing small changes to the existing training data or generating synthetic data from the existing dataset to increase its size. We applied data augmentation techniques, including horizontal and vertical flipping, random rotation, color jitter, and shearing to our training dataset.

### 3.3. Pre-Training

Recent advancements in the field of computer vision have seen transformers achieve state-of-the-art results, surpassing CNNs in various tasks. Despite this success, it is important to note that transformer architectures tend to be more data hungry than CNNs, necessitating a large amount of training data for optimal performance. Given the inherent challenge of data scarcity in medical imaging, transfer learning emerges as a promising approach to enhance the effectiveness of transformer models. By pre-training transformer models on large datasets and subsequently fine-tuning them on smaller, domain-specific datasets, notable improvements in performance have been observed. It outperformed the CNN architectures in many computer vision tasks such as object detection [27,37], semantic segmentation [38,39,40], and image classification [41]. All the models used in this study were pre-trained with the ImageNet dataset and then transfer-leaned to our oral cancer image dataset. In this study, the transformer-based models were pre-trained with the ImageNet dataset.

### 3.4. Experiments Setup

Code implementation was made on PyTorch (pytorch.org, accessed on 1 December 2023) and all the training was performed on the high-performance computing platform of the University of Arizona. We trained all the networks in this study with cross-entropy loss and the Adam optimization algorithm [42]. To enhance the robustness of the training set, we applied data augmentation techniques, which including random rotation, vertical and horizontal flipping, color jitter and shearing, before training each network. Each training session utilized an initial learning rate of 10^−3^, which decayed 10-fold every 50 epochs. The total number of epochs was set to 180, and a batch size of 32 was employed. We saved the models with the best validation accuracy. In all experiments in this study, we performed 5-fold cross-validation.

### 3.5. The Experiments Results

In this study, the VIT and Swin Transformer models are compared with two classic convolutional neural networks, VGG19 and ResNet50. The performance of these models were compared through sensitivity, specificity, positive prediction value, negative prediction value and accuracy. Table 1 lists the 5-fold cross-validation sensitivity, specificity, positive prediction value, negative prediction value, and accuracy of all models on our dataset. It can be seen from the table that the Swin Transformer model performs best with a sensitivity of 0.905, a specificity of 0.870, a PPV of 0.870, a NPV of 0.905, and an accuracy of 0.887. The accuracy of the Swin Transformer model is 2.3%, 4.2% and 3.5% higher than that of the VIT, ResNet50 and VGG19, respectively. The sensitivity of the Swin Transformer model is 3.3%, 5.7% and 4.1% higher than that of the VIT, ResNet50 and VGG19, respectively. The specificity of the Swin Transformer model is 2.4%, 2.8% and 2.9% higher than that of the VIT, ResNet50 and VGG19, respectively.

## 4. Discussion

Despite their impressive performance, transformer-based models also have some limitations. Although the interpretability and explainability of CNNs based models are not very good, transformer-based models are even harder to interpret, making it challenging to understand the predictions, because interpretable models are essential in medical applications to gain insights into decision-making processes and to build trust among healthcare professionals and patients. Another limitation is that transformer-based models typically require more computational resources compared to CNNs due to their self-attention mechanism and large parameter space. This increased computational overhead may pose challenges for real-time or resource-constrained applications in cancer image analysis. In our previous study, we have performed extensive tests with our previous CNN models for oral cancer image analysis. We will perform more validation assessments and analyses with transformer-based models in the future since they have shown promising performance with the preliminary tests.

## 5. Conclusions and Future Work

In the realm of global health, the escalating prevalence of oral cancer necessitates innovative diagnostic solutions. This study explores the potential of Vision Transformers (ViT) and Swin Transformers in mobile-based oral cancer image classification. Benchmark results reveal that the pre-trained Swin Transformer model achieved an 88.7% accuracy in binary classification, outperforming the ViT model by 2.3% and surpassing the conventional VGG19 and ResNet50 CNN models, which achieved 85.2 and 84.5% accuracy. These findings underscore the capability of Transformer-based architectures, particularly the Swin Transformer, in advancing state-of-the-art oral cancer image analysis.

While this study demonstrates the effectiveness and potential of transformer-based oral cancer classification, future research could focus on exploring real-world integration feasibility, optimizing transfer learning strategies, and investigating multimodal approaches given the availability of a multimodal oral cancer dataset we previously collected. Additionally, exploring the reliability, interpretability, and trustworthiness of transformer-based models for oral cancer diagnosis is crucial. Addressing these aspects in future research can contribute to the broader applicability of transformer-based architectures, fostering advancements in global healthcare.

## Figures and Tables

**Table 1:** The 5-fold cross-validation sensitivity, specificity, positive prediction value, negative prediction value and accuracy of the ViT, the Swin Transformer, VGG19, and ResNet50.

5-Fold Cross-Validation Results	Sensitivity	Specificity	PPV	NPV	Accuracy
VGG19	0.864	0.841	0.839	0.866	0.852
ResNet50	0.848	0.842	0.838	0.853	0.845
ViT	0.872	0.856	0.853	0.875	0.864
Swin Transformer	0.905	0.870	0.870	0.905	0.887

## Data Availability

The dataset is not currently public available due to regulation restriction.
